# In vitro evaluation of defined oligosaccharide fractions in an equine model of inflammation

**DOI:** 10.1186/1746-6148-9-147

**Published:** 2013-07-22

**Authors:** Johannes Cornelis Vendrig, Luc Edgar Coffeng, Johanna Fink-Gremmels

**Affiliations:** 1 Department of Veterinary Pharmacology, Pharmacotherapy and Toxicology, Faculty of Veterinary Medicine, Utrecht University, P.O. box 80152, 3508, TD Utrecht, The Netherlands; 2 Department of Public Health, Erasmus MC, University Medical Center Rotterdam, P.O. box 3000, 2040, CA Rotterdam, The Netherlands

**Keywords:** Horse, PBMC, Oligosaccharide, Inflammation, Immunomodulation, GOS, FOS, AOS, Cytokines, Bayesian hierarchical linear regression

## Abstract

**Background:**

Dietary supplementation with oligosaccharides has been proven to be beneficial for health in several mammalian species. Next to prebiotic effects resulting in a modulation of gut micro biota, immunomodulatory effects of oligosaccharides have been documented *in vivo*. Supplementation with defined oligosaccharide fractions has been shown to attenuate allergic responses and enhance defensive immune responses. Despite the accumulating evidence for immunomodulatory effects, very limited information is available regarding the direct mechanism of action of oligosaccharides. This study aims to elucidate the effects of selected oligosaccharide fractions on the lipopolysaccharide (LPS) induced inflammatory response in equine peripheral blood mononuclear cells (PBMCs). We investigated three different products containing either galacto-oligosaccharides (GOS) alone, a combination of GOS with fructo-oligosaccharides (FOS), and a triple combination of GOS and FOS with acidic oligosaccharides (AOS), at different concentrations. These products have been used in an identical composition in various previously published *in vivo* experiments. As the selected oligosaccharide fractions were derived from natural products, the fractions contained defined amounts of mono- and disaccharides and minor amounts of endotoxin, which was taken into account in the design of the study and the analysis of data. Acquired data were analysed in a Bayesian hierarchical linear regression model, accounting for variation between horses.

**Results:**

Exposing cultured PBMCs to either GOS or GOS/FOS fractions resulted in a substantial dose-dependent increase of tumour necrosis factor-α (TNF-α) production in LPS challenged PBMCs. In contrast, incubation with GOS/FOS/AOS resulted in a dose-dependent reduction of both TNF-α and interleukin-10 production following LPS challenge. In addition, incubation with GOS/FOS/AOS significantly increased the apparent PBMC viability, indicating a protective or mitogenic effect. Furthermore, mono- and disaccharide control fractions significantly stimulated the inflammatory response in LPS challenged PBMCs as well, though to a lesser extent than GOS and GOS/FOS fractions.

**Conclusions:**

We found distinct immunomodulating effects of the investigated standardised oligosaccharide fractions, which either stimulated or suppressed the LPS induced inflammatory response in PBMCs. Both scenarios require additional investigation, to elucidate underlying modulatory mechanisms, and to translate this knowledge into the clinical application of oligosaccharide supplements in foals and other neonates.

## Background

During the past decade, numerous *in vivo* studies in humans (and experimental animals) have reported beneficial effects of dietary supplementation with oligosaccharides derived from natural products such as milk, fruits and vegetables. The original goal of supplementing infant formulas with oligosaccharide fractions was to mimic prebiotic effects of human milk oligosaccharides in non-breastfed infants. Several oligosaccharide fractions were synthesised as possible surrogates of human milk oligosaccharides. Short chain galacto-oligosaccharides (GOS) are oligomers of lactose (degree of polymerization (dp) 2–6), produced by elongating lactose using β-galactosidase enzymes [1]. GOS is applied either alone or in combination with long-chain fructo-oligosaccharides (FOS), using a GOS:FOS ratio of 9:1. FOS fractions are acquired by removing the short-chain fructans from chicory inulin, resulting in fructan mixtures with terminal glucose monomers and a minimal dp of 22 [1]. During the last few years, additions of methylated pectin- derived acidic oligosaccharides (AOS) to infant formulas were investigated as well, mostly combined with both GOS and FOS (GOS:FOS:AOS ratio of 9:1:2) [1]. The prebiotic properties of these commercially produced GOS/FOS and GOS/FOS/AOS fractions have been proven in various studies [2-5].

Moreover, immunomodulatory properties of GOS and combinations of both GOS/FOS and GOS/FOS/AOS have been documented *in vivo*. Dietary supplementation with these oligosaccharide fractions was shown to be beneficial for both allergic immune responses [6-10] and defensive immune responses following viral or bacterial challenge [11-13]. Furthermore, there is evidence that early supplementation with GOS/FOS (with or without AOS) lowers the incidence of infections in human infants [14,15]. In most of these studies, the effects of oligosaccharides on the intestinal microbiotica were discussed as the primary mode of action of the administered oligosaccharides. In contrast, very limited information is available concerning any direct immunomodulatory effects of these oligosaccharide fractions and the underlying mechanisms*.* Eiwegger et al. [16] reported that human milk-derived oligosaccharides and plant-derived oligosaccharides (low-molecular-weight fucoidan) affect the cytokine production and activation of unchallenged cord blood derived T cells *in vitro*. In a more recent study by the same group, the *in vitro* incubation of unchallenged human cord blood mononuclear cells with low concentrations (10–100 μg/ml) of AOS or GOS combined with FOS did not result in an alteration of cytokine production, whereas incubation with similar concentrations of acidic human milk-derived oligosaccharides did significantly induce the production of interferon-γ and interleukin-10 [17]. The latter study also provides *in vitro* evidence for epithelial transport of prebiotic oligosaccharides, enabling direct contact between oligosaccharides and cells of the immune system.

Neonatal foals possess limited defence mechanisms, in particular due to the impermeability of the equine placenta to maternal immunoglobulins. Consequently, newborn foals are strongly dependent on the transfer of immunoglobulins through the uptake of colostrum [18]. Moreover, similar to newborns of other mammalian species, both innate and adaptive immune responses are immature at the time of delivery [19]. Dietary supplementation of oligosaccharides would be one of the possible options to improve the development of the immune system and consequently lower the incidence of infections in foals, which are often life-threatening. However, up to now no research has been published regarding immunomodulatory effects of oligosaccharides in the horse, neither *in vivo* nor *in vitro*.

In this study, we investigated the effects of defined oligosaccharide fractions on the inflammatory response in equine peripheral blood mononuclear cells (PBMCs) following a standardised lipopolysaccharide (LPS) challenge. We chose to use equine PBMCs as a readily available representative model of immune cells in this animal species.

## Methods

### Animals and sample collection

Twelve healthy, adult, Dutch warm blood horses (K.W.P.N. studbook) were sampled in total during this study. PBMCs from five horses were used for experiments with oligosaccharide fractions, four horses were sampled for control experiments with glucose/lactose solutions, and PBMCs of three horses were used for cell viability assays. Experiments could not all be executed in the same horses, due to the large amounts of PBMCs required to investigate all fractions and concentrations in triplicate (at least). Adult horses had to be used as blood donors, as large volumes of blood were needed to isolate sufficient amounts of PBMCs for these first explorative experiments in the horse; withdrawing larger volumes of blood from neonatal foals is restricted by ethical concerns. From each horse, 250 ml of blood was collected by jugular venipuncture directly into sterile heparinised blood collection tubes^a^. Blood samples were kept cooled and PBMC isolation was started within 1 hour after collection. All experimental procedures were approved by the committee of ethical considerations regarding animal experiments of Utrecht University (DEC Utrecht).

### PBMC isolation

Blood samples were diluted 1:1 in sterile PBS^b^ containing 2 mM EDTA^c^ and subsequently layered over Ficoll- Paque™ plus^d^. After centrifugation (400× *g*, 30 minutes at room temperature), PBMCs were pipetted from the Ficoll layer and washed twice in PBS/EDTA. PBMCs were resuspended in RPMI 1640 Medium^b^ supplemented with 2 mM glutamine^b^, 100 IU/ml penicillin^b^, 100 μg/ml streptomycin^b^, and 10% horse serum (prepared in our own laboratory according to standard procedures). PBMCs were counted using trypan blue and resuspended to a density of 4*10^6^ cells/ml medium. Following storage overnight at 4°C to attenuate possible stimulatory effects of the applied Ficoll, PBMCs were seeded in 24 well plates at a density of 4*10^6^ cells/ml medium/well.

### Cell culture experiments

After seeding the PBMCs in 24 well plates, the plates were incubated for 2 hours at 37°C and 5% CO_2_. After this, the plates were centrifuged for 10 minutes at 400× *g* before refreshing the medium without removing PBMCs. The experiments were started by pre-incubating the PBMCs for 2 hours with supplemented RPMI containing different concentrations of oligosaccharide fractions (including blank controls, i.e. supplemented RPMI without extra additions). After pre-incubation, plates were centrifuged again and the medium was replaced with medium containing 0 or 1 μg/ml LPS (*Escherichia coli* O111:B4)^c^ combined with different concentrations of oligosaccharide fractions (including blank controls). Plates were placed in the incubator for another 4 hours, after which samples for ELISA were collected and stored at −80°C. Thus, there was a total incubation time of 6 hours for all investigated conditions. All different incubations were performed in triplicate or in quadruplicate for each horse. Additional experiments were performed following the same protocol with different concentrations of glucose and lactose, to account for the fact that the oligosaccharide fractions- especially GOS- contain considerable amounts of these mono- and disaccharides.

We investigated the effects of three different standardised oligosaccharide fractions with known immunomodulatory properties *in vivo,* using oligosaccharide concentrations ranging from 0.5% to 2.0%. The exact composition of all 2% incubations is summarized in Table 1 (1% and 0.5% solutions were obtained by diluting 1:1 in supplemented RPMI). The applied GOS fraction (Vivinal GOS syrup^e^, dp 2–6) consisted of approximately 45% GOS, 14% glucose, 16% lactose and 25% water. GOS was investigated separately, combined with FOS (Raftiline HP^f^, dp ≥ 23, approximately 96.5% FOS and 3.5% maltodextrine) in a 9:1 ratio, and combined with both FOS and AOS^g^ (dp 1–20, approximately 85.5% AOS, 7.25% monomers and 7.25% moisture) in a 9:1:2 ratio. Ratio’s and diluting factors were based on oligosaccharide contents of the used products. Thus, all 2% oligosaccharide solutions contained 20 mg/ml oligosaccharides and, in addition, defined amounts of mono- and disaccharides. The control incubations with glucose^c^ and lactose^c^ were chosen to correspond with the glucose and lactose concentrations of the GOS fraction, as this was the oligosaccharide fraction with the highest concentrations of mono- and disaccharides (see Table 1). The effects of glucose/lactose controls were investigated to eventually discriminate between effects of the oligosaccharide preparations and possible effects of glucose and lactose, which are present in the oligosaccharide fractions as well. All experiments in this study were performed using the same batch of GOS, FOS and AOS, of which the composition was analysed and stated by the manufacturers.

**Table 1:** Composition of the applied oligosaccharide fractions and glucose/lactose controls (per 10 ml of medium)

**Incubation**	**Oligosaccharides**	**Glucose**	**Lactose**
GOS 2%	200 mg GOS	62.22 mg	71.11 mg
GOS/FOS 2%	180 mg GOS, 20 mg FOS (+0.73 mg maltodextrin)	56.00 mg	64.00 mg
GOS/FOS/AOS 2%	150 mg GOS, 16.67 mg FOS (+0.60 mg maltodextrin), 33.33 mg AOS	46.67 mg (+2.83 mg monomers)	53.33 mg
glucose/lactose, corresponding with GOS 2%	-	62.22 mg	71.11 mg

### ELISA

Protein levels of tumour necrosis factor-α (TNF-α) and interleukin-10 (IL-10) were measured by means of ELISA on the cell culture supernatants, using Duo set® ELISA Development System for equine TNF-α and equine IL-10^h^. Standard operating procedures of the manufacturer were followed, applying all required buffers and solutions in the form provided by the manufacturer^h^. The detection limits of the ELISAs were 15.625 pg/ml (TNF-α) and 156.25 pg/ml (IL-10), respectively.

### Cell viability assessment

To investigate possible influence of the applied incubation mixtures on cell viability, CCK-8^c^ viability assays were performed according to manufacturer’s instructions^c^. Prior to viability assessment, cell culture experiments were carried out identical to the experiments which are described above, with the exception that cells were seeded and incubated in 96 well plates (at a density of 8*10^5^ cells/200 μl/well, similar to experiments in terms of cells per ml medium and cm^2^ surface).

### Endotoxin assay

All applied solutions (as described in Table 1) were tested for endotoxin contamination using a Limulus Ambocyte Lysate endotoxin assay according to manu-facturer’s instructions^i^. The selected standard curve (determining the range of detection) ranged from 0.1-1 Endotoxin Unit (EU)/ml.

### Data analysis and statistical methods

Data were analysed by means of Bayesian hierarchical linear regression, assuming a normal distribution of the log-transformed ELISA data. A hierarchical approach was taken to account for variation between horses. PBMC responses were allowed to vary between horses with regard to general PBMC reactivity (random intercept), and PBMC reactivity specifically against incubation with LPS, GOS, FOS, and AOS (random slopes). Analyses were performed in a Bayesian framework, using JAGS, a program for analysis of Bayesian models using Markov Chain Monte Carlo (MCMC) simulation (version 3.2.0) [20]. Simulations in JAGS were set up and analysed in R (version 2.14.2) [21], using packages *rjags* (version 3–5) [22] and *R2jags* (version 0.03-06) [23]. Posterior distributions were simulated based on uninformative prior distributions (normal distributions with mean 0 and standard deviation 100 for parameter means; inverse gamma distributions with mean 1 and variance 10,000 for parameter variances; inverse scaled Wishart distribution for the variance-covariance matrix of random effects). Bayesian credible intervals (BCI) for parameter estimates were based on the 2.5% and 97.5% percentiles of posterior distributions. Posterior distributions were simulated by means of four Markov chains, each consisting of 1 million Monte Carlo samples (saving only every 50th sample). The first half of all samples was discarded for burn-in, allowing the model to converge. Model convergence was assessed according to Gelman and Rubin’s convergence diagnostic, the potential scale reduction factor [24].

Differences are stated significant, based on the calculated 95% BCI. In the results section, significant differences are quantified by percentages. Not all significant differences are quantified in the results section. An overview of all data and the corresponding 95% BCI’s is given in an Additional file 1, annexed to the manuscript.

## Results

### Oligosaccharide fractions dose-dependently modulate TNF-α production in unchallenged PBMCs

In Table 2, TNF-α and IL-10 production is illustrated after incubation of PBMCs with oligosaccharide fractions (including blank controls). All fractions significantly increased TNF-α production by PBMCs compared to blank controls and did so at all concentrations. Dose-dependent effects were seen for all three oligosaccharide fractions. For GOS, the dose-dependent increase was only significant after incubation with 2% GOS compared to incubation with 1% GOS (43% increase). For GOS/FOS, the dose-dependent increase of TNF-α production was significant through the whole concentration range (1% vs. 0.5%: 110% increase, 2% vs. 1%: 101% increase). For GOS/FOS/AOS, a significant increase of TNF-α production was evident when comparing the 1% incubation with the 0.5% incubation (81% increase). In contrast, PBMCs incubated with 2% GOS/FOS/AOS produced significantly lower amounts of TNF-α compared to PBMCs incubated with 1% GOS/FOS/AOS (54% reduction).

Production of IL-10 in unchallenged PBMCs was not significantly influenced by oligosaccharide fractions in comparison with blank controls.

**Table 2:** TNF-α and IL-10 production in unchallenged PBMCs incubated with oligosaccharide fractions

	**TNF- α (pg/ml)**			**IL-10 (pg/ml)**		
**Incubation**	**Point estimate**	**Lower bound**	**Upper bound**	**Point estimate**	**Lower bound**	**Upper bound**
Blank controls	**12.6**	9.8	15.7	**187.2**	156.2	222.5
GOS 0.5%	19.7 *	16.5	23.3	**183.1**	152.8	217.7
GOS 1.0%	24.9 *	21.1	29.3	**184.2**	153.5	219.2
GOS 2.0%	35.5 *^#^	30.2	41.6	**179.8**	149.6	214.2
GOS/FOS 0.5%	30.2 *	25.7	35.4	**185.5**	154.8	221.4
GOS/FOS 1.0%	63.4 *^#^	54.1	74.2	**190.2**	160.0	225.7
GOS/FOS 2.0%	127.5 *^#^	108.6	149.5	**186.0**	154.5	221.4
GOS/FOS/AOS 0.5%	73.6 *	62.9	86.4	**205.2**	173.8	242.5
GOS/FOS/AOS 1.0%	133.5 *^#^	113.7	156.6	**214.2**	181.6	252.4
GOS/FOS/AOS 2.0%	61.4 *^#^	52.4	72.2	**186.8**	157.3	221.0

Mean protein concentrations of TNF-α and IL-10 (pg/ml) and 95% Bayesian Credible Intervals for unchallenged PBMCs incubated with oligosaccharide fractions (including blank controls). Significant differences between oligosaccharide fractions and blank controls are marked with an asterisk (*) Significant dose-dependent differences (1.0% vs. 0.5% and 2.0% vs. 1.0%) are marked with a hash (^#^).

### Oligosaccharide fractions dose-dependently modulate cytokine production in LPS challenged PBMCs

Table 3 illustrates TNF-α and IL-10 production in LPS challenged PBMCs after pre-incubation and co-incubation with the applied oligosaccharide fractions (including incubations with LPS alone). The TNF-α response in PBMCs incubated with LPS alone was significantly increased with 13,793% (138-fold) in comparison with blank controls. Incubation with 0.5% solutions of all three oligosaccharide fractions resulted in significantly higher TNF-α concentrations compared to incubation with LPS alone. A dose-dependent increase of TNF-α production was observed for GOS and GOS/FOS within the entire concentration range. When comparing 1% incubations with 0.5% incubations, the increase of TNF-α production was 36% for GOS and 53% for GOS/FOS. For GOS, incubation with 2% solutions resulted in an additional significant increase of TNF-α production compared to 1% solutions (55% increase), whereas for GOS/FOS this increase was not significant. In contrast to GOS and GOS/FOS, incubation with increasing concentrations of GOS/FOS/AOS resulted in a dose-dependent reduction of TNF-α production in LPS challenged PBMCs (1% vs. 0.5%: 22% reduction; 2% vs. 1%: 69% reduction). Moreover, TNF-α production in LPS challenged PBMCs incubated with GOS/FOS/AOS 2% was significantly lower compared to incubation with LPS alone (41% reduction).

The IL-10 response in PBMCs incubated with LPS alone was significantly increased with 49% in comparison with blank controls. Whereas both GOS and GOS/FOS incubations did not significantly alter the IL-10 response following LPS stimulation, incubation with LPS and GOS/FOS/AOS 0.5% did increase IL-10 production significantly in comparison with incubation with LPS alone (35% increase). Incubation with higher concentrations of GOS/FOS/AOS resulted in a dose-dependent reduction of the LPS induced IL-10 response in PBMCs (1% vs. 0.5%: 28% reduction, 2% vs. 1%: 33% reduction). Moreover, IL-10 production in LPS challenged PBMCs incubated with GOS/FOS/AOS 2% was significantly lower compared to incubation with LPS alone (35% reduction).

**Table 3:** TNF-α and IL-10 production in LPS challenged PBMCs incubated with oligosaccharide fractions

	**TNF- α (pg/ml)**			**IL-10 (pg/ml)**		
**Incubation**	**Point estimate**	**Lower bound**	**Upper bound**	**Point estimate**	**Lower bound**	**Upper bound**
LPS 1 μg/ml	**1749.4**	1495.2	2050.8	**278.1**	236.0	328.7
LPS + GOS 0.5%	3655.5 *	3115.0	4285.5	**293.8**	248.4	347.6
LPS + GOS 1.0%	4989.0 *^#^	4251.4	5843.0	**268.8**	227.5	316.7
LPS + GOS 2.0%	7754.3 *^#^	6607.8	9090.6	**265.6**	225.0	313.6
LPS + GOS/FOS 0.5%	3601.1 *	3074.8	4221.7	**344.1**	290.9	407.5
LPS + GOS/FOS 1.0%	5530.3 *^#^	4712.6	6483.4	**307.4**	260.6	362.9
LPS + GOS/FOS 2.0%	6721.1 *	5733.0	7879.3	**264.0**	223.9	311.7
LPS + GOS/FOS/AOS 0.5%	4242.9 *	3608.3	4969.1	374.3 *	317.0	442.3
LPS + GOS/FOS/AOS 1.0%	3317.6 *^#^	2832.7	3893.3	**269.6 **^**#**^	227.9	318.3
LPS + GOS/FOS/AOS 2.0%	1029.7 *^#^	874.8	1205.9	180.7 *^#^	149.0	216.6

Mean protein concentrations of TNF-α and IL-10 (pg/ml) and 95% Bayesian Credible Intervals for LPS challenged PBMCs incubated with oligosaccharide fractions (including incubations with LPS alone). Significant differences between oligosaccharide fractions and LPS alone are marked with an asterisk (*). Significant dose-dependent differences (1.0% vs. 0.5% and 2.0% vs. 1.0%) are marked with a hash (^#^).

### Oligosaccharide fractions affect the TNF-α response significantly more profoundly compared to mono- and disaccharide controls

**Figure 1:**
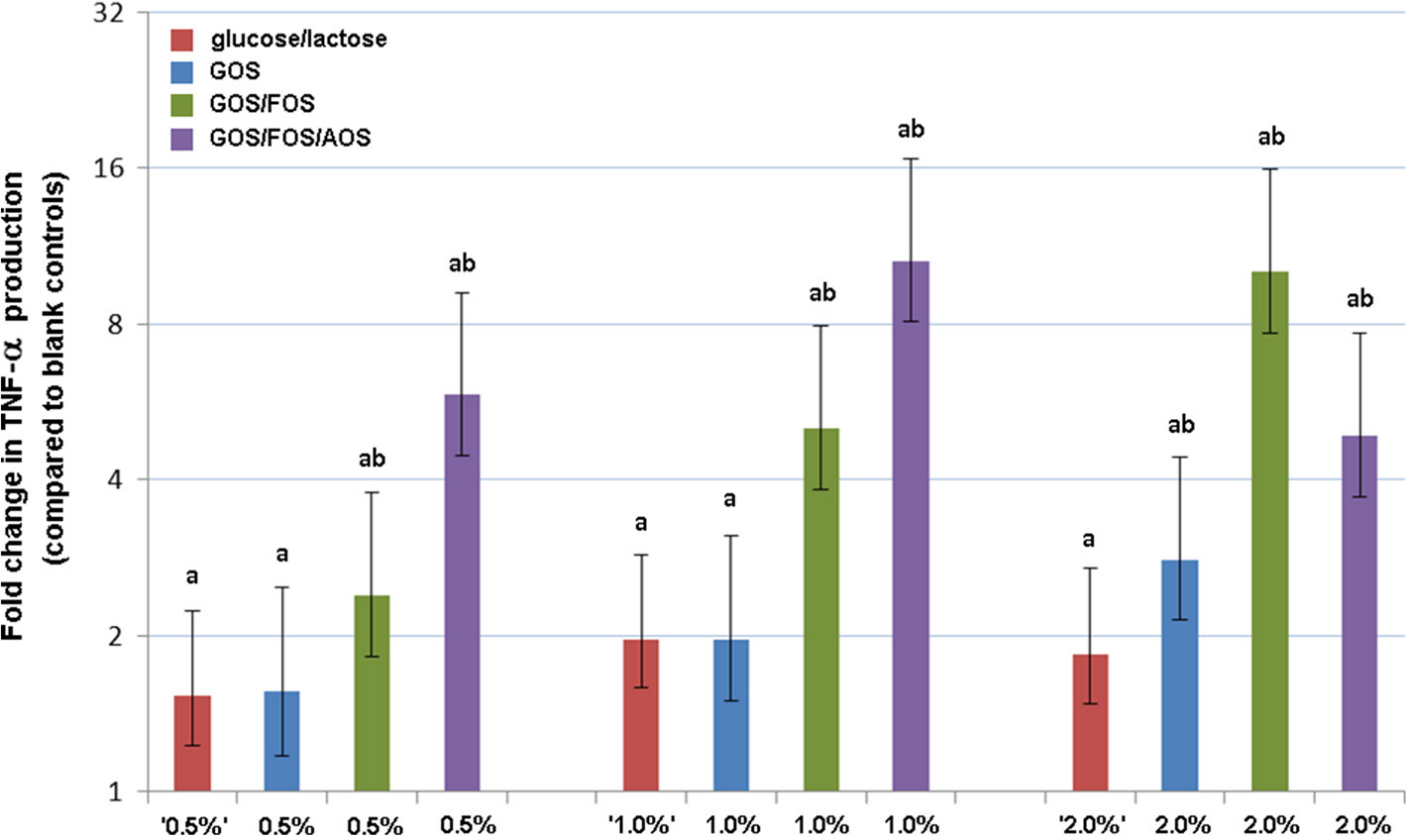
**Comparison of the effects of oligosaccharide fractions vs. glucose/lactose controls on TNF-α response in unchallenged PBMCs.** Relative TNF-α production compared to blank controls and 95% Bayesian Credible Intervals are given for all investigated incubations, including glucose/lactose controls (glucose/lactose ‘x%’ meaning corresponding with glucose/lactose concentrations of x% GOS). Significant differences are marked with lower case letters. a: Significant difference between oligosaccharide or glucose/lactose fractions and blank controls. b: Significant difference between oligosaccharide fractions and corresponding glucose/lactose controls.

To account for the mono- and disaccharide contents of the applied oligosaccharide preparations, effects of these oligosaccharide preparations were compared with effects of corresponding glucose/lactose concentrations in the same model. A comparison of the effects of oligosaccharide preparations and glucose/lactose controls on the TNF-α response in unchallenged PBMCs is given in Figure 1.

Incubation of PBMCs with medium containing glucose and lactose resulted in significantly higher TNF-α production in comparison with blank controls (increase of 53%, 97%, and 84% for glucose/lactose solutions resembling 0.5%, 1% and 2% GOS, respectively). However, no significant dose-dependent effects were detected among the glucose/lactose incubations.

A comparison with the GOS incubations revealed that incubation of unchallenged PBMCs with 2% GOS resulted in significantly more TNF-α production compared to the corresponding glucose/lactose controls (52% increase), whereas effects of the 0.5% and 1% GOS and corresponding glucose lactose incubations did not differ significantly. For GOS/FOS and GOS/FOS/AOS, all effects on TNF-α production were significantly more profound compared to the effects of corresponding glucose/lactose controls.

**Figure 2:**
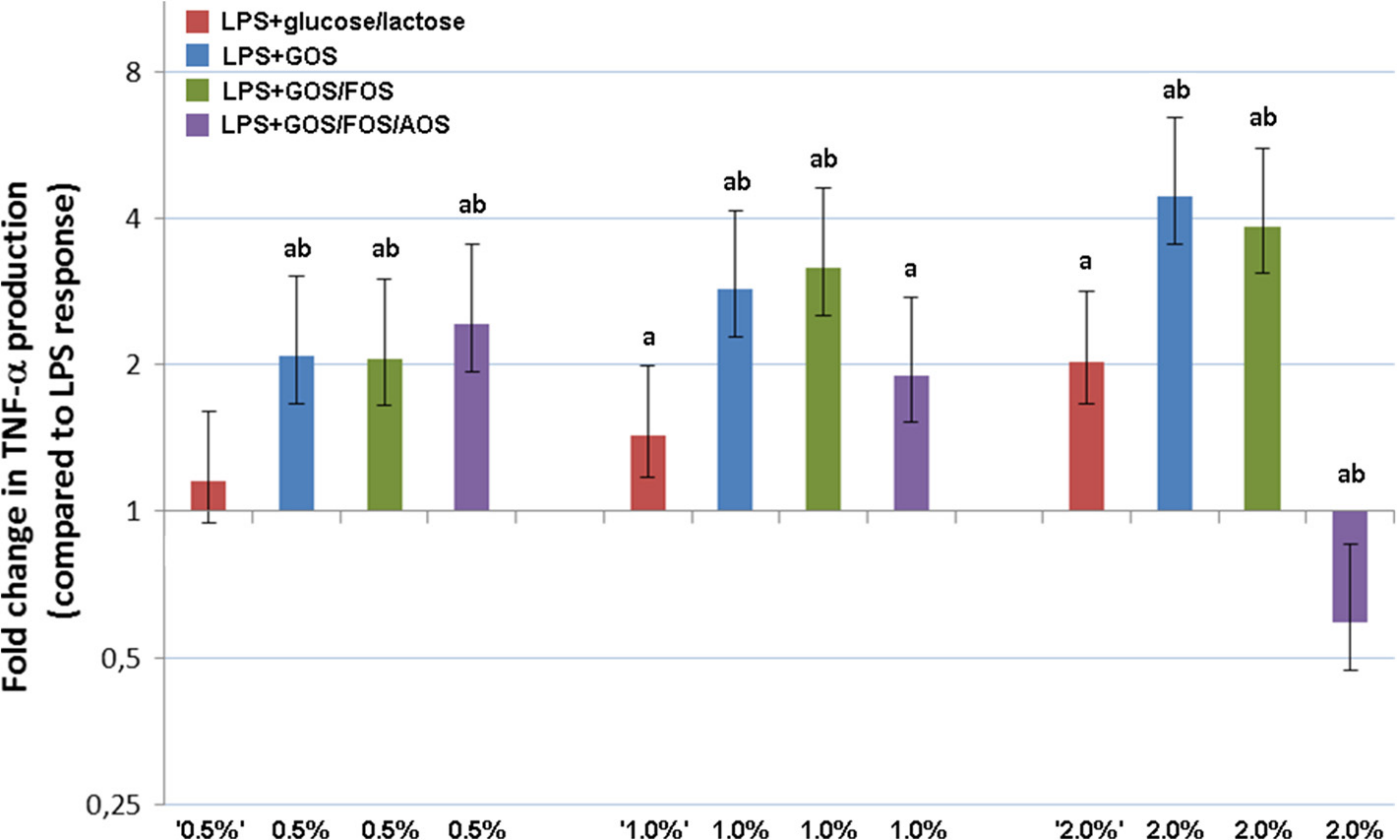
**Comparison of the effects of oligosaccharide fractions vs. glucose/lactose controls on TNF-α response in LPS challenged PBMCs.** Relative TNF-α production compared to PBMCs incubated with 1 μg/ml LPS and 95% Bayesian Credible Intervals are given for all investigated incubations, including glucose/lactose controls (glucose/lactose ‘x%’ meaning corresponding with glucose/lactose concentrations of x% GOS). Significant differences are marked with lower case letters. a: Significant difference between oligosaccharide or glucose/lactose fractions and LPS alone. b: Significant difference between oligosaccharide fractions and corresponding glucose/lactose controls.

Effects of oligosaccharide preparations and glucose/lactose controls on the TNF-α response in LPS challenged PBMCs are compared in Figure 2. Whereas incubation of PBMCs with glucose/lactose resembling 0.5% GOS did not alter LPS induced TNF-α production significantly, the glucose/lactose solutions corresponding with 1% and 2% GOS enhanced the LPS induced TNF-α production dose-dependently (1% vs. 0.5%: 24% increase; 2% vs. 1%: 42% increase).

Differences between effects of the applied oligosaccharide preparations and glucose/lactose controls were evident through the entire concentration range. For all investigated GOS and GOS/FOS concentrations, TNF-α production was significantly higher after incubation with the oligosaccharide fraction compared to the corresponding glucose/lactose concentrations. In contrast, for GOS/FOS/AOS a dose-dependent decrease in TNF-α production was evident. LPS challenged PBMCs incubated with GOS/FOS/AOS 2% produced significantly less TNF-α compared to corresponding glucose/lactose controls (71% reduction).

A comparison of the effects of oligosaccharide preparations and corresponding glucose/lactose concentrations on IL-10 production could not be executed, as IL-10 concentrations which were measured in the glucose/lactose experiments were mostly below the detection limit of the ELISA.

### Incubation of PBMCs with GOS/FOS/AOS significantly enhances cell viability

**Figure 3:**
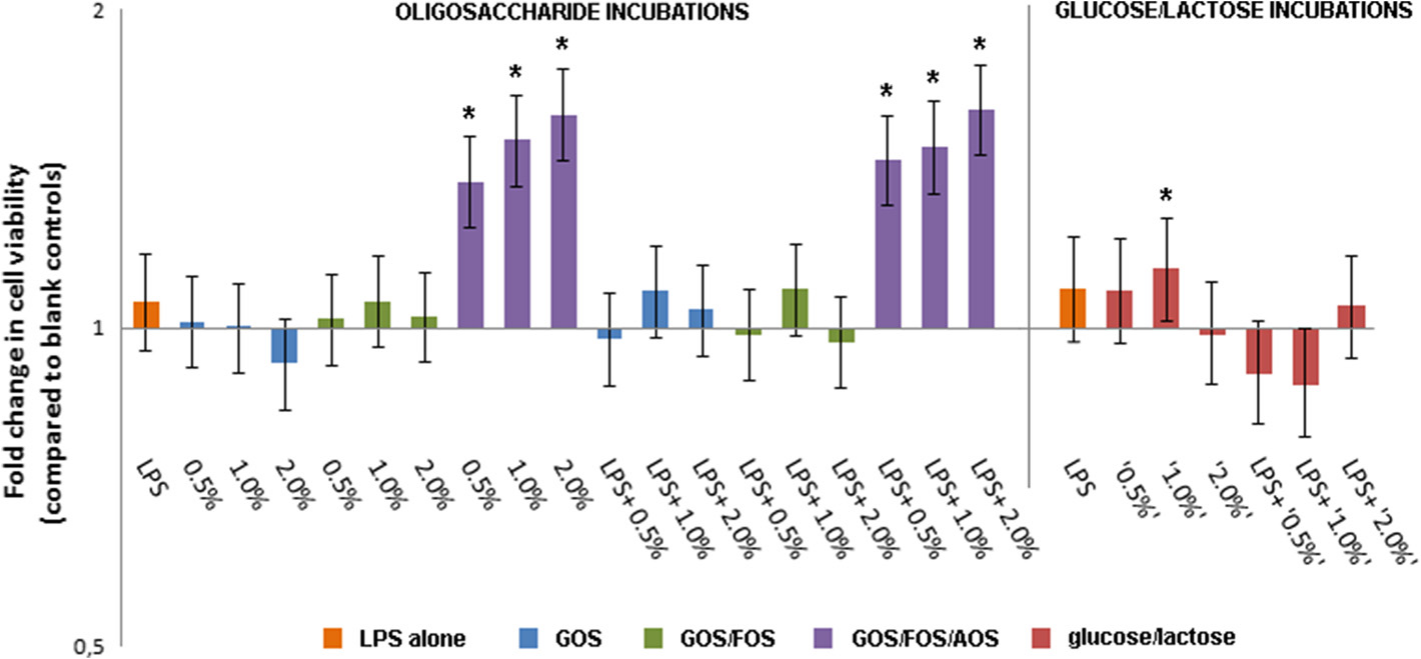
**Results for the CCK-8 viability assay.** Cell viability is given for all investigated incubations, relative to cell viability as determined in blank controls, including 95% Bayesian Credible Intervals. Significant increases of cell viability are marked with an asterisk (*).

Results for the CCK-8 viability assays are illustrated in Figure 3. The investigated incubations did not significantly reduce cell viability compared to blank controls. However, all investigated GOS/FOS/AOS incubations significantly increased the apparent cell viability (ranging from 38-61% increase). Besides this, a mild (14%) but significant increase of cell viability was observed for glucose/lactose concentrations resembling 1% GOS.

### Minor endotoxin contamination of the applied oligosaccharide fractions

According to the performed bioassay, all oligosaccharide solutions contained measurable concentrations of endotoxin. For the 2% solutions, the endotoxin concentrations (mean values ± standard deviation) were estimated at 0.24 ± 0.02 EU/ml (GOS), 1.06 EU/ml (GOS/FOS, based on measurements in 1% solution: 0.53 ± 0.07 EU/ml) and 1.92 EU/ml (GOS/FOS/AOS, based on measurements in 1% solution: 0.96 ± 0.07 EU/ml). The glucose and lactose solutions did not contain measurable amounts of endotoxin (lower detection limit of the assay was 0.1 EU/ml).

## Discussion

### Individual oligosaccharides exhibit distinct immunomodulatory properties

Based on our results, we can conclude that both GOS and GOS/FOS fractions dose-dependently enhanced the pro-inflammatory response by LPS challenged equine PBMCs. In contrast, after incubation with 2% GOS/FOS/AOS the LPS induced inflammatory response was dose-dependently reduced. This indicates that with increasing the AOS concentration in the incubations, the stimulatory effects of both GOS and GOS/FOS were eventually overruled by the suppressive effects of AOS in this model. Suppression of the cytokine response was not caused by cytotoxic properties of AOS. On the contrary, PBMC viability was significantly increased in all GOS/FOS/AOS incubations compared to blank incubations, suggesting a protective or mitogenic effect. In conclusion, the reduced cytokine production by PBMCs incubated with GOS/FOS/AOS was not the result of a reduced number of viable cells, as lower amounts of cytokines were produced by a higher number of cells.

We cannot exclude that a direct interaction between AOS and LPS influenced the LPS response in PBMCs incubated with GOS/FOS/AOS. Binding of LPS by AOS in the gut lumen might even contribute to the documented beneficial effects of oligosaccharide fractions containing AOS in *in vivo* studies.

Though we observed modulatory effects of mono- and disaccharides on the LPS induced inflammatory response in our model, these effects were less pronounced and overshadowed by the immunomodulating effects of the investigated oligosaccharide fractions. To our knowledge, literature regarding the underlying mechanism of action of mono- and disaccharides, which enhance the LPS induced inflammatory response, is lacking. However, our results are in line with a recently published study, which reported that high glucose conditions enhance the TNF-α and IL-6 response following LPS challenge [25].

### Endotoxin contamination is not responsible for the enhancement of the LPS response by oligosaccharide fractions

As PBMCs are extremely sensitive to bacterial endotoxin, it cannot be excluded that the significant increase of TNF-α production in unchallenged PBMCs after incubation with the applied oligosaccharide fractions was caused by the low endotoxin concentrations in these natural products. Although it is not possible to give an exact concentration based on the results obtained with the Limulus assay, it can be assumed that the degree of endotoxin contamination ranged from 1.20- 19.20 ng/ml for the 2% oligosaccharide solutions. This estimate is based on the information of the manufacturer^c^ of the applied LPS (1 ng LPS = 5–10 EU). We challenged the PBMCs with 1000 ng/ml LPS, as preliminary experiments with this PBMC model indicated that this concentration already results in a maximum TNF-α response after 4 hours of incubation. As 1.20- 19.20 ng/ml is almost negligible compared to a concentration of 1000 ng/ml, it can be assumed that this low endotoxin contamination cannot be held accountable for the enhancement of the LPS response by the oligosaccharide fractions in our model. Moreover, the enhancement of the TNF-α response in LPS challenged cells was not proportional to the mild stimulatory effects in unchallenged PBMCs.

However, endotoxin contamination might play a role in the reduction of the LPS response after incubation with 2% GOS/FOS/AOS. In this solution, the measured endotoxin concentration was the highest (approximately 10–19 ng/ml) and we cannot exclude that endotoxin tolerance was induced during the PBMC pre-incubation period, suppressing the response after the actual challenge [26]. On the other hand, the 1% solution of GOS/FOS/AOS (approximately 5–10 ng/ml endotoxin) already caused a significant reduction of the LPS response in comparison with the 0.5% GOS/FOS/AOS solution, whilst the 2% GOS/FOS solution (approximately 6–11 ng/ml endotoxin) induced a significant increase of the LPS response compared with the 1% GOS/FOS solution. Hence, distinct modulatory effects of the AOS fraction remain plausible.

### Direct effects of oligosaccharides

Previously performed *in vivo* studies often ascribe immunomodulatory effects of dietary oligosaccharides to their prebiotic properties. However, the results of this study support the hypothesis of direct modulatory effects of oligosaccharides, independent of the intestinal micro biota. Though this notion is also supported by other *in vitro* studies [16,17], the method of action behind direct immunomodulation by oligosaccharides remains unclear. Carbohydrate (glycan)- binding receptors expressed on the surface of intestinal epithelial cells and antigen presenting cells may be involved in modulation of the immune response by sugar molecules [27]. In addition, next to modulation of Toll-like receptor (TLR) signalling in the gastrointestinal tract due to the changes in the gut flora, oligosaccharides may directly modulate TLR- signalling. For instance, hyaluronan oligosaccharides have been shown to directly mediate inflammatory responses via TLR-4 [28,29]. As known TLR-4 ligands such as LPS display structural similarity with the investigated oligosaccharides, other oligosaccharides may influence TLR-4 signalling as well.

As of yet, research with oligosaccharides mainly involved direct intervention studies in human infants or in experimental (rodent) animal species as surrogates for human infants [6-15]. One *in vivo* study in adult horses demonstrated beneficial effects of prebiotic short-chain fructo-oligosaccharides, preventing disruption of the intestinal flora in the equine hindgut in case of an abrupt change of diet composition [30]. Concerning immunomodulation by oligosaccharides in the horse, no data have been published to our knowledge. The data obtained from the present study confirm the activity of heterologous oligosaccharides in horses and are in line with the very few published *in vitro* studies with human cell culture models [16,17].

The most notable finding was the divergence between the immuno-activation of PBMCs by GOS and GOS/FOS preparations and the apparent suppressive effect of AOS. This was an unexpected outcome and indicates that forthcoming experimental protocols should include conditions with AOS only. In the current experiments we applied the (commercially available) combinations of oligosaccharides used in previous studies, and hence AOS was not tested separately.

## Conclusions

In conclusion, this study provides evidence that oligosaccharides from different sources have distinct direct immunomodulatory properties. We showed that both GOS and FOS dose-dependently stimulated the LPS induced inflammatory response in equine PBMCs, and that AOS dose-dependently suppressed the production of both TNF-α and IL-10 following an LPS challenge. Such an activation or suppression of the immune system could be beneficial *in vivo*, depending on the clinical context. Future research into dietary supplementation of specific oligosaccharide fractions in foals and underlying immunomodulatory mechanisms could lead to the development of oligosaccharide supplements in foals, to support a rapid maturation of the immune competence in the first phase of life, hence protecting them from early, often life-threatening infections.

## Availability of supporting data

The data supporting the results of this article are included within the article and its Additional file 1 (annexed file with model parameters estimates and 95% Bayesian Credible Intervals).

## Endnotes

^a^BD Vacutainer Systems, Plymouth, United Kingdom

^b^Lonza, Basel, Switzerland

^c^Sigma- Aldrich, St. Louis, USA

^d^GE Healthcare, Waukesha, USA

^e^FrieslandCampina Domo, Zwolle, The Netherlands

^f^Orafti, Wijchen, The Netherlands

^g^Danone Research, Wageningen, The Netherlands

^h^R&D Systems, Minneapolis, USA

^i^Genscript, Piscataway, USA

## Abbreviations

AOS: Acidic oligosaccharides; dp: degree of polymerization; IL: Interleukin; FOS: Fructo-oligosaccharides; GOS: Galacto-oligosaccharides; LPS: Lipopolysaccharide; PBMC: Peripheral blood mononuclear cell; TLR: Toll- like receptor; TNF: Tumour necrosis factor.

## Competing interests

The authors declare that they have no competing interests.

## Authors’ contributions

Conceived and designed the study: JF, JV. Collected materials and performed experiments: JV. Analysed the data: JV, LC. Wrote the manuscript: JV. Reviewed and revised the manuscript: JF, JV, LC. All authors read and approved the final manuscript.

## Supplementary Material

Additional file 1 **TNF-alpha protein: model parameter estimates and 95% Bayesian Credible Intervals.** IL-10 protein: model parameter estimates and 95% Bayesian Credible Intervals. CCK-8 assay: model parameter estimates and 95% Bayesian Credible Intervals. Click here for file
